# Addressing Two Systemic Bottlenecks in Global Poultry Production: A Review of Non-Destructive Pre-Incubation Freshness and In-Ovo Sex Detection

**DOI:** 10.3390/ani16152370

**Published:** 2026-08-03

**Authors:** Qian Yan, Zengrui Wu, Hanzhi Chen, Dayou Zhou, Yingli Wang, Xiao Zhou

**Affiliations:** 1 Ningbo Academy of Agricultural Sciences, Ningbo 315040, China; yqyq@webmail.hzau.edu.cn; 2 College of Engineering, Huazhong Agricultural University, Wuhan 430070, China; ruiw@webmail.hzau.edu.cn (Z.W.); hanzhichen@webmail.hzau.edu.cn (H.C.); dayou@webmail.hzau.edu.cn (D.Z.)

**Keywords:** hatching egg, non-destructive detection, pre-incubation freshness, in ovo sexing, industrial implementation, poultry sustainability

## Abstract

The $300-billion global poultry industry, a cornerstone of food security, faces two interconnected systemic challenges: pre-incubation quality degradation wastes up to 15% of incubation capacity on non-viable eggs, while post-hatch male chick culling results in the annual euthanasia of 7 billion day-old layer chicks. Existing non-destructive detection research remains fragmented, treating pre-incubation freshness assessment and in ovo sexing as disconnected domains and creating a persistent lab-to-field gap that blocks industrial translation. This review establishes a unified process-centric framework covering the full detection workflow, and critically evaluates state-of-the-art sensing modalities across the entire hatching cycle. The analysis demonstrates that implementation bottlenecks do not reside in laboratory classification accuracy, which frequently exceeds 90% under controlled conditions, but in cross-batch generalizability, embryonic biosafety assurance, and high-throughput online operation. A hierarchical detection strategy with closed-loop traceability is outlined to bridge the lab-to-field gap. This work provides a structured roadmap to improve both economic efficiency and ethical sustainability of global poultry production.

## Simple Summary

The global poultry industry supplies affordable protein to billions of people, yet it faces two longstanding wasteful and ethically concerning practices. First, up to 15% of incubation space is wasted on eggs that will never hatch due to quality decline before incubation. Second, approximately 7 billion newly hatched male layer chicks are culled annually, as they do not lay eggs and are unsuitable for efficient meat production. Past research has studied these two issues separately, slowing the translation of laboratory advances to real farm use. This review combines the two topics into a unified, workflow-based framework and evaluates all major detection technologies across the full hatching cycle. The analysis finds that the main barrier to industrial use is no longer laboratory accuracy, but poor stability across variable production conditions, high maintenance costs, and uncertain embryo safety. A tiered detection strategy is proposed to balance speed, accuracy, and cost, supporting more efficient and humane poultry production.

## 1. Introduction

The global poultry industry is an irreplaceable pillar of global food security, annually supplying over 130 million tons of eggs and 120 million tons of poultry meat to feed billions worldwide. At the core of this $300-billion industry, commercial hatcheries act as the critical production bottleneck: even a 1% improvement in hatching efficiency translates to billions of dollars in annual economic gains and substantial reductions in resource waste [1]. However, two interconnected systemic challenges have plagued the industry for decades, limiting its long-term sustainability and social legitimacy. First, pre-incubation quality degradation squanders up to 15% of valuable incubation capacity on non-viable eggs that will never hatch. Second, post-hatch male chick culling results in the annual euthanasia of an estimated 7 billion day-old male layer chicks globally—a practice that has triggered widespread public concern and impending regulatory bans across Europe and other regions [2]. These intractable problems demand transformative technological solutions that address both economic inefficiency and animal welfare concerns simultaneously.

Non-destructive detection technologies have emerged as the most promising pathway to resolve these dual challenges. Unlike static table egg quality assessment, hatching egg inspection requires tracking dynamic biological processes unfolding over weeks. Pre-incubation internal quality, fertility status, and storage freshness fundamentally define the upper limit of embryonic development potential, while incubation brings progressive changes in vascularization, metabolism, and sexual differentiation that generate measurable external signals. For industrial deployment, hatcheries require a seamless, minimally invasive pipeline that identifies unviable or male eggs early without disrupting the delicate embryonic microenvironment [3]. This imposes a fundamental constraint: any viable technology must simultaneously balance four mutually competing metrics—early detection, non-destructiveness, high throughput, and robustness to real-world variability [4]. Eggshell color, weight, storage duration, orientation, and batch-to-batch differences introduce significant baseline variability that often renders laboratory-validated differences unreliable in industrial settings.

A critical structural flaw undermines the existing research landscape: pre-incubation freshness evaluation and incubation-stage in ovo sexing have historically been studied as entirely disconnected domains. This siloed approach creates a fatal blind spot. Freshness-related changes—including water loss, air cell enlargement, and albumen thinning—directly alter the physical and chemical properties of eggs, and thereby modify the very signals used for later in ovo sex detection [5,6]. Conventional manual candling, the de facto industry standard, is a subjective process with accuracy rarely exceeding 70% before incubation day 10, and cannot reliably capture these subtle, interconnected changes [7]. While advanced techniques including spectral imaging, machine vision, and thermal imaging have been developed over the past three decades, their development has proceeded in isolation, with no unifying framework to guide integration across the full hatching cycle [8,9].

**Figure 1:**
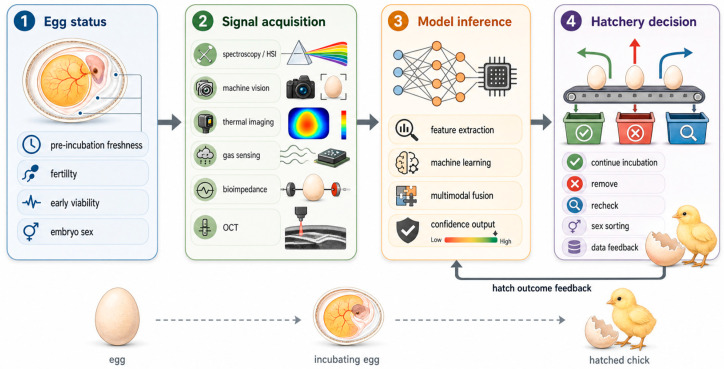
Overall workflow for non-destructive detection of hatching eggs.

As depicted in Figure 1, this review proposes a unified, process-centric framework organized around the complete workflow of “pre-incubation screening—incubation-stage discrimination—sorting decision”. This architecture decomposes the entire detection process into four sequential, interdependent modules: intra-egg state transitions, signal acquisition, feature modeling, and hatchery decision implementation. By standardizing comparison of detection windows, signal strengths, and equipment requirements across both freshness and sex assessment, this framework enables systematic evaluation of technology performance across the entire hatching cycle rather than at isolated time points.

Despite decades of extensive research, three critical, unaddressed limitations continue to prevent widespread industrial adoption of non-destructive detection technologies. First, very few studies have systematically examined how pre-incubation storage conditions affect the performance of in ovo sex detection systems, despite clear evidence that storage-induced changes profoundly alter egg properties [10,11,12]. Second, the vast majority of research prioritizes peak laboratory accuracies achieved under highly controlled conditions while neglecting real-world confounding variables, creating a persistent “lab-to-field chasm” [13]. Third, no widely accepted comparative framework exists to evaluate technologies across the full hatching cycle, with existing reviews focusing narrowly on single techniques or applications rather than providing a holistic roadmap for industrial implementation [14].

This review addresses these gaps by presenting a unified workflow-based framework that integrates freshness and sex detection into a continuous, end-to-end process. State-of-the-art sensing modalities are systematically evaluated across the entire hatching cycle, the remaining technical and operational barriers to industrialization are identified, and a hierarchical detection strategy with closed-loop traceability is proposed to bridge the gap between laboratory research and commercial application. By synthesizing decades of fragmented research into a coherent, actionable framework, this work provides a clear roadmap for future research and accelerates translation of promising technologies to enhance both the economic efficiency and ethical sustainability of global poultry production.

## 2. Materials and Methods

This review adopts a systematic narrative synthesis approach to integrate evidence on non-destructive pre-incubation freshness assessment and in ovo sex detection for poultry hatching eggs, establishing a unified workflow-based analytical framework covering the full hatching cycle.

Literature was retrieved from six authoritative databases (Web of Science Core Collection, ScienceDirect, PubMed, IEEE Xplore, CNKI and Wanfang Data) from database inception to June 2026. Search terms spanned three dimensions: research subject, technical modality and core objective, including combinations of “hatching egg sexing”, “non-destructive sex detection”, “hyperspectral imaging”, “computer vision”, “pre-incubation freshness” and “embryo viability assessment” in both English and Chinese. Reference lists of highly cited reviews and landmark studies were manually screened via snowballing to supplement potentially omitted relevant research.

After duplicate removal using EndNote X9 (Clarivate Analytics, Philadelphia, PA, USA), eligible studies were identified through three sequential screening stages: title screening, abstract screening and full-text evaluation. Inclusion criteria were original research focusing on non-destructive detection of poultry hatching eggs, with clear technical principles, complete experimental designs and quantifiable performance metrics. Studies were excluded if they applied destructive detection methods, targeted adult poultry or processed poultry products, consisted of purely commentary without empirical data, or were duplicate publications with obvious logical flaws. A total of 72 eligible studies were finally included, covering all mainstream technical categories: spectral detection, machine vision, thermal imaging, volatile organic compound analysis, bioimpedance, optical coherence tomography and multimodal fusion.

A standardized data extraction template was applied to collect core information including study characteristics, sample attributes, detection performance, model validation strategies and industrial applicability. Methodological quality was appraised based on experimental design rigor, sample representativeness and performance validation standardization. Technologies were classified by working principle to clarify their core mechanisms and evolution trajectories, and horizontally compared across key dimensions of detection accuracy, throughput, operational complexity, equipment cost and embryonic biosafety, to identify industrialization barriers and propose practical implementation pathways.

## 3. Signal Fundamentals and Technical Framework of Non-Destructive Hatching Egg Detection

### 3.1. Biological Basis of Detection Signals

The core challenge of non-destructive hatching egg detection lies in establishing robust, reproducible correlations between dynamic intra-egg state transitions and externally measurable physical or chemical signals. During storage and incubation, hatching eggs undergo continuous, quantifiable changes in egg weight, air cell volume, albumen viscosity, allantoic fluid composition, embryonic temperature, and eggshell porosity. Among these, well-documented linear relationships between weight loss rate, air cell size, and hatching success have long served as the foundational basis for industrial incubation management practices [15,16]. Although these traditional metrics are primarily derived from offline measurements, they provide critical guidance for rational selection of non-destructive sensing modalities.

A unique and underappreciated characteristic of hatching eggs as detection targets is that individual eggs undergo sequential physical, chemical, and physiological transformations throughout both storage and incubation phases. The pre-incubation phase is primarily associated with internal quality degradation, fertility status, and embryonic viability, while the incubation phase is marked by progressive emergence of signals linked to vascularization, metabolic gas release, and sexual differentiation. Critically, detection technologies that focus solely on the existence of measurable statistical differences risk failing to distinguish the biological origins of these signals—whether they arise from albumen, yolk, germinal disk, embryonic vasculature, or environmental confounding factors. This mechanistic ambiguity is the single largest contributor to poor cross-batch generalizability of laboratory-validated models.

### 3.2. Classification and Mechanisms of Main Sensing Modalities

Optical signals remain the most widely adopted detection modality in existing research, owing to their non-contact nature and high throughput potential. Visible–near-infrared (Vis-NIR) spectroscopy captures differences in water, protein, lipid, blood, and embryonic tissue composition through changes in light absorption and scattering properties. Hyperspectral imaging (HSI) extends this capability by integrating spatial information with spectral responses, enabling targeted identification of germinal disks, blood vessels, yolk, and albumen regions within a single egg [17,18]. Digital imaging and thermal imaging, by contrast, emphasize morphological and thermal distribution patterns, making them particularly suitable for detecting air cell boundaries, vascular textures, and embryonic contours [19,20].

When spectral or imaging signals alone are insufficient to explain observed classification differences, volatile organic compounds (VOCs), bioimpedance, and optical coherence tomography (OCT) provide complementary signals that probe metabolic release, electrical properties, and microstructural localization, respectively [21,22,23]. Each modality exhibits unique strengths and inherent limitations: optical methods offer high throughput and non-contact operation, while gas and electrical techniques provide more direct insights into embryonic metabolic activity. No single modality currently satisfies all four core industrial requirements (early detection, non-destructiveness, high throughput, robustness), necessitating multi-modal fusion approaches for comprehensive assessment.

### 3.3. Algorithmic Foundations for Classification and Modeling

At the algorithmic level, principal component analysis (PCA), linear discriminant analysis (LDA), and partial least squares discriminant analysis (PLS-DA) remain the predominant methods for small-sample spectral modeling. PLS-DA is particularly suitable for handling collinear spectral data with limited sample sizes (*n* < 200) and provides relatively interpretable coefficient plots to identify informative wavelengths, but it is highly sensitive to batch shifts and requires strict standardization of acquisition conditions. PCA, as an unsupervised dimensionality reduction technique, is primarily used for exploratory data analysis and outlier removal rather than final classification, as it does not incorporate label information.

Support vector machines (SVM), random forests (RF), and artificial neural networks (ANN) are employed to address nonlinear classification problems. SVM performs well on medium-sized datasets (200 < *n* < 1000) with clear class boundaries and is less prone to overfitting than shallow ANNs, but its computational cost increases significantly with sample size. RF excels at handling noisy data and automatically evaluating feature importance, making it ideal for multi-modal feature fusion, but it may struggle with highly imbalanced datasets. Convolutional neural networks (CNN) and deep learning architectures enable automatic feature extraction from images or spectral matrices, eliminating the need for manual feature engineering. However, CNNs require large annotated datasets (*n* > 5000) to achieve stable performance, suffer from poor interpretability (the “black box” problem), and are computationally intensive to train and deploy on edge devices.

Comparative studies on public hatching egg datasets have shown that for small-sample spectral fertility detection, PLS-DA outperforms SVM and CNN in terms of both accuracy and computational efficiency; for large-scale blood vessel image-based in ovo sexing, CNN achieves 5–10% higher accuracy than traditional machine learning methods but requires 10–20 times more training data [24,25,26]. Crucially, however, model complexity cannot compensate for poor signal acquisition stability. For highly heterogeneous targets such as hatching eggs, factors including incubation day, eggshell color, genetic strain, storage duration, egg orientation, and incubation batch typically exert a far greater influence on model generalizability than the specific algorithm employed. This indicates that rigorous experimental design and standardized signal acquisition protocols are more critical for robust performance than selection of advanced modeling techniques.

### 3.4. Mechanistic Validation Techniques and Critical Appraisal

Beyond spectroscopy and imaging, structural imaging and developmental monitoring studies provide essential mechanistic references for signal interpretation. Nuclear magnetic resonance (NMR) microimaging enables visualization of extra-embryonic blood flow and internal compositional changes, while volume-selective NMR allows quantitative measurement of diffusion and relaxation parameters within the egg. Magnetic resonance imaging (MRI), computed tomography (CT), and ultrasound further characterize embryonic development from the perspectives of soft tissue morphology, skeletal development, and gross anatomical structure [27,28,29,30,31]. More recently, laser speckle contrast imaging has been demonstrated to visualize early extra-embryonic vasculature through intact eggshells, while visible light transmission and HSI have been applied to monitor embryonic developmental progression [32,33,34,35].

From a critical perspective, these mechanistic techniques remain largely confined to laboratory settings. Most have low throughput, require expensive specialized equipment, and are incompatible with high-speed commercial sorting lines. Furthermore, the vast majority of mechanistic validation studies are conducted on single genetic strains under strictly controlled conditions, so their conclusions may not generalize to the diverse egg populations found in commercial hatcheries. Without scalable mechanistic validation, classification models remain vulnerable to confounding from non-biological sources of signal variation.

## 4. Non-Destructive Detection of Pre-Incubation Freshness and Internal Quality

### 4.1. Technical Objectives and Unique Requirements

The technical objective of pre-incubation freshness detection is to identify eggs with degraded internal quality, abnormal fertility status, or reduced hatching potential either before they are loaded into incubators or during the earliest stages of incubation. Unlike freshness evaluation of table eggs, hatching egg inspection must additionally account for embryonic safety and subsequent hatching outcomes. Consequently, it is insufficient to predict only conventional metrics such as Haugh unit or yolk index; assessments must also include discrimination between fertile and infertile eggs, early dead embryos, and viable embryos [5].

**Figure 2:**
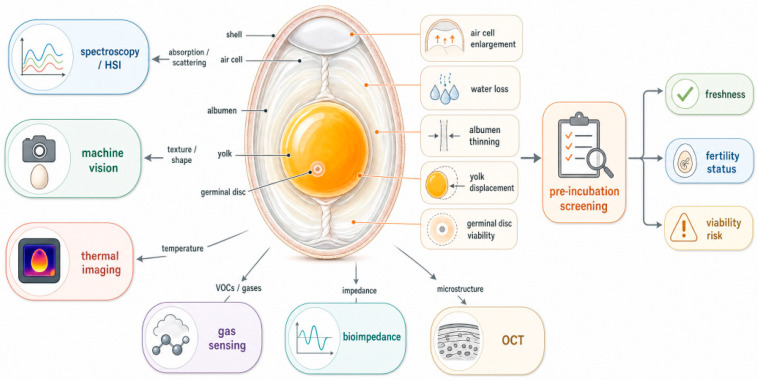
Signal Transformation Pathways for Pre-Incubation Hatching Egg Freshness and Internal Quality Detection.

A common and pervasive misconception is that pre-incubation hatching egg detection is equivalent to table egg freshness evaluation. However, the primary goal of hatching egg production is not to assign a static quality score, but to minimize wasted incubation resources by identifying unsuitable eggs at the earliest possible stage. As illustrated in Figure 2, air cell enlargement, albumen thinning, yolk displacement, and changes in germinal disk viability translate into measurable changes in spectral absorption or scattering, image texture, thermal field distribution, gas emission, electrical impedance, and tomographic structure. The complex interactions between fertility status, early embryonic viability, storage duration, and eggshell physical properties necessitate comprehensive discussion of all relevant detection modalities.

### 4.2. Hyperspectral Imaging for Fertility and Viability Assessment

HSI represents the most extensively researched technology for pre-incubation fertility detection. One landmark study employed Vis-SWNIR transmission imaging to assess fertility in unincubated chicken eggs, extracting spectral features from the 400–760 nm and 900–1700 nm ranges and classifying samples using LDA, ANN, and SVM models, with classification accuracies approaching 90% for certain models [17]. Another line of research combined hyperspectral imaging with relevance vector machines for non-destructive hatching egg inspection, demonstrating that combined spectral-spatial information in the 400–1000 nm range provides valuable evidence for fertility discrimination [18].

The significance of these findings lies not in the peak accuracy achieved by any single model, but in the fundamental demonstration that structural and compositional differences exist in unincubated eggs that are detectable via spectroscopic techniques. The availability of public HSI datasets has further lowered barriers to experimental reproducibility, enabling consistent comparison of fertility status, egg shape, and structural parameters across identical sample sets [36].

### 4.3. Effects of Storage Conditions on Detection Performance

The effects of storage conditions on detection performance require dedicated consideration in freshness detection systems, as storage-induced biological changes directly alter the physical and chemical signals used for non-destructive testing. Prolonged storage (≥7 days) causes albumen thinning and water migration, which increases light transmittance in the 700–900 nm spectral range by 15–25% and shifts the baseline of NIR spectra, leading to a 10–18% decrease in the accuracy of PLS-DA-based fertility detection models. Storage temperature fluctuations (±2 °C) accelerate air cell enlargement and create heterogeneous thermal gradients across the eggshell, reducing the accuracy of thermal imaging-based air cell detection by 8–12%. High relative humidity (>75%) during storage inhibits water loss and weakens the correlation between egg weight loss and freshness, making egg-parameter-based storage time prediction models unreliable. Prolonged storage also alters germinal disk position and intra-egg structural organization, increasing the variability of OCT-based germinal disk localization results by 20–30% [10,11,12].

These findings indicate that Haugh unit, yolk index, or single morphological parameters only capture partial aspects of egg quality and cannot replace comprehensive assessment of germinal disk position, air cell dynamics, gas exchange, and ultimate hatching outcomes [37,38,39]. Critically, storage conditions also modify the performance of in ovo sexing technologies. Studies have shown that eggs stored for more than 10 days exhibit a 12–20% reduction in the accuracy of day-4 blood vessel-based sex detection, as delayed embryonic development weakens sex-related vascular morphology differences. Similarly, storage-induced changes in eggshell porosity alter VOC emission rates, reducing the discriminative power of gas-based sex detection methods by 15–25%. Short-term pre-incubation heating has been shown to partially restore embryonic viability in stored hatching eggs and mitigate some of these detection performance degradations [40].

### 4.4. Complementary Detection Modalities

Effective freshness and storage duration monitoring relies on detection of continuous, time-dependent signals rather than discrete measurements. Studies on non-destructive storage duration control based on egg parameters have shown that features including egg shape, mass, and air cell size can be used to develop accurate storage time prediction models [41]. Non-invasive micro-test technology enables direct monitoring of oxygen respiration patterns in stored eggs, while diode laser spectroscopy targets free oxygen and water vapor, integrating gas exchange measurements into fertility and incubation status assessment systems [42,43]. Acoustic resonance technique has detected a decrease in resonant frequency in the 100–500 Hz range at approximately 100 h of incubation, which has been correlated with albumen dehydration and mechanical changes at the shell-membrane interface [44].

Thermal imaging and machine vision offer distinct advantages of non-contact operation, high speed, and ease of integration into existing production lines. Water loss during incubation drives air cell enlargement, making air cell boundaries and thermal field distribution valuable diagnostic features. Studies on air cell dynamics monitoring using thermal imaging have employed CNN and BP neural networks for air cell region segmentation, achieving detection mean average precision up to 99.85% [19]. The established relationships between eggshell thickness, translucency, density, geometric parameters, weight loss, and hatching rate further suggest that machine vision and physical parameter measurement can serve as low-cost entry points for freshness risk screening [45,46,47,48].

### 4.5. Industrial Implementation Challenges and Critical Appraisal

Collectively, these studies demonstrate that pre-incubation freshness detection has evolved into a multi-modal landscape. Spectroscopic and hyperspectral methods are best suited for capturing internal compositional and structural differences; machine vision, thermal imaging, and egg parameter measurement are most applicable to high-throughput primary screening; and gas exchange and blood flow-related signals provide valuable insights into embryonic viability. The technical characteristics and application conditions of representative studies are summarized in Table 1.

From a critical standpoint, nearly all existing pre-incubation detection studies suffer from three shared limitations. First, performance is almost exclusively validated on single batches of eggs from a single genetic strain, with no independent external validation across different flocks or production environments. Second, most studies report only classification accuracy, with very few tracking hatching rate, chick quality, or long-term developmental outcomes to verify that detection procedures do not harm embryos. Third, the vast majority of experimental setups use horizontally oriented eggs under static conditions, which do not reflect the vertical orientation and continuous movement of eggs on commercial production lines. These methodological gaps are primary contributors to the performance drop observed during industrial deployment.

## 5. Non-Destructive and Minimally Invasive Detection of Embryonic Sex During Incubation

### 5.1. Fundamental Trade-Off Between Detection Earliness and Signal Robustness

The fundamental challenge of embryonic sex detection lies in the detection time window. Earlier detection yields greater economic benefits through reduced incubation space requirements and eliminates the need for post-hatch male chick culling, thereby addressing critical animal welfare concerns. However, sex-related differences are extremely weak in early development, making detection signals highly susceptible to confounding by eggshell color, embryonic orientation, and batch variability. Conversely, later detection benefits from stronger signals associated with vascular development, metabolic differentiation, and gonadal maturation, but at the cost of diminished economic and ethical returns [49,50].

**Figure 3:**
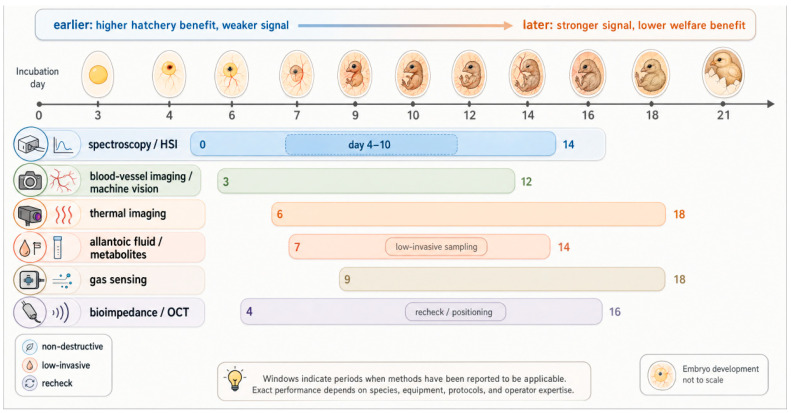
Inherent trade-off between detection earliness and signal robustness for embryonic sex identification across different incubation stages.

Consequently, in ovo sexing studies cannot be evaluated solely on the basis of classification accuracy; they must also report incubation day at detection, embryonic safety, detection speed, and validation methodology. As illustrated in Figure 3, the complexity of technical approaches for in ovo sexing arises from this inherent trade-off between detection earliness and signal robustness. Early stage differences in blood vessel morphology and metabolism are too weak to be reliably detected, making models highly vulnerable to interference from eggshell color, acquisition position, and embryonic orientation. Mid-to-late stage signals are significantly more robust, but their utility is compromised by the reduced economic and ethical benefits of later detection.

### 5.2. Spectroscopic and Hyperspectral Imaging Approaches

Spectroscopy and hyperspectral imaging represent the most intensively researched directions for in ovo sexing. One pioneering study on early chicken embryo sex identification using hyperspectral imaging collected data across 0–12 days of incubation, developing PLS-DA, SVM, and ANN models using spectral features in the 600–900 nm range around day 10, and demonstrating that sex-related differences can be captured through combined spectral-spatial features [51]. Subsequent studies applied visible–near-infrared hyperspectral technology to chicken egg sexing across 0–14 days of incubation, identifying informative feature bands in the 400–1000 nm range and comparing the performance of PLS-DA, SVM, and neural network models [24].

Collectively, these studies confirm that sex-specific information is indeed present in early optical signals. However, its reliable extraction requires rigorous control of acquisition position, systematic sample stratification, and independent external validation. Comparative studies have demonstrated that model performance is significantly affected by measurement location (blunt vs. sharp end), illumination mode (diffuse reflectance vs. transmission), and eggshell color (white vs. brown strains). Consequently, reported accuracies are meaningless unless accompanied by detailed descriptions of acquisition conditions [52,53,54].

### 5.3. Multi-Modal Fusion Strategies

Multi-modal fusion represents the most promising strategy to address the challenge of weak early-stage signals. One study developed an RF-D-S spectral-image information fusion approach that combined visible–near-infrared transmission spectra with machine vision features, performing decision-level fusion using random forests and Dempster-Shafer evidence theory. This approach achieved an accuracy of approximately 88.00% at day 4 of incubation and identified characteristic wavelengths at 587.14, 588.04, 588.49, and 589.39 nm [55]. Another study combined blood vessel texture features with a GA-BP neural network to capture embryonic sex differences from image texture patterns, demonstrating that vascular morphology provides indirect information for sex identification [20].

However, multi-modal systems typically require additional sensors, more complex synchronized acquisition hardware, and higher maintenance costs, which limits their widespread industrial adoption. The incremental accuracy gain from fusion also varies widely across studies, with some reporting gains of 5–10% and others reporting no statistically significant improvement, likely due to differences in signal complementarity and experimental design.

### 5.4. Complementary Detection Technologies and Validation Methods

Beyond spectroscopy and imaging, fluorescence spectroscopy, Raman spectroscopy, VOC analysis, OCT, and electrical detection form a complementary portfolio of sex identification technologies. Two-wavelength fluorescence spectroscopy has been shown to capture strong sex-related responses at specific incubation days [56]. Raman and fluorescence-Raman approaches aim to identify sex-specific markers from molecular vibration information in blood or embryonic tissue [57,58]. Solid-phase microextraction/gas chromatography-mass spectrometry and active VOC sampling convert metabolic differences between male and female embryos into measurable odor profiles [22,59,60].

Molecular and cytological methods should be strictly positioned as validation and calibration tools, not as primary non-destructive detection technologies. PCR, real-time PCR, loop-mediated isothermal amplification, recombinase polymerase amplification, and yolk proteomics can provide highly reliable sex determination or mechanistic insights, but they typically require invasive sampling, sample destruction, or laboratory-based workflows [21,61,62,63,64,65]. Flow cytometry discriminates chicken embryo sex based on differences in sex chromosome DNA content, while fluorescence in situ hybridization and triple-primer PCR serve as validation tools for W chromosome or Phasianidae-specific sex markers [66,67].

### 5.5. Critical Considerations for Performance Evaluation

Reported accuracies in sex detection literature are meaningless unless interpreted in conjunction with incubation day, genetic strain, eggshell color, acquisition position, sample size, and gold standard validation methodology. Comparing high accuracies achieved in late incubation directly with early-stage minimally invasive detection results leads to significant overestimation of the industrial value of certain methods. Similarly, failure to report hatching rates and chick quality can result in underestimation of the potential adverse effects of detection procedures on embryonic safety. The key differences among relevant studies are summarized in Table 2.

### 5.6. Cross-Species Comparative Analysis

While most research has focused on chicken hatching eggs, global waterfowl (duck, goose) and specialty poultry (quail, turkey) production accounts for over 30% of total poultry output, creating significant demand for species-specific non-destructive detection technologies. This section provides a structured comparison across five major poultry species.

#### 5.6.1. Chicken (Gallus Domesticus)

Chicken eggs are the most extensively studied model, with the broadest range of validated detection technologies. White-shelled layer strains generally yield the highest detection accuracies for both freshness and sexing, due to high light transmittance and relatively low phenotypic variability. Brown-shelled strains consistently show 5–15% lower detection accuracy across all optical methods, due to stronger light absorption by eggshell pigments [53]. Broiler strains exhibit larger egg size and higher yolk lipid content, which alter spectral baselines and require model recalibration.

#### 5.6.2. Duck

For duck hatching eggs, Vis-NIR spectroscopy combined with CNN models has achieved 84.65–93.36% accuracy for in ovo sexing between days 0 and 8 of incubation, outperforming traditional machine learning methods by 10–15% [25]. However, duck eggs have thicker eggshells and higher yolk lipid content than chicken eggs, which reduces light transmittance in the 900–1700 nm range and requires optimized optical path designs. Hyperspectral imaging has also been applied to detect fertility and freshness in duck eggs, with accuracies exceeding 88% for unincubated fertility detection.

#### 5.6.3. Goose

Goose egg detection faces additional challenges due to their larger size, thicker eggshells, and longer incubation period. Recent studies have used thermal imaging to monitor air cell development in goose eggs, achieving 92% accuracy for early dead embryo detection at day 7 of incubation. Very few studies have addressed in ovo sexing in goose eggs, and no validated non-destructive method is currently available for commercial use, representing a major research gap.

#### 5.6.4. Quail

Quail eggs are characterized by their small size and highly variable eggshell coloration, which complicates optical detection. Studies have shown that multi-position Vis-NIR spectroscopy can achieve 85% accuracy for quail egg fertility detection before incubation, while blood vessel imaging-based in ovo sexing reaches 82% accuracy at day 3 of incubation [43]. Transportation vibration has a particularly significant impact on quail egg quality, increasing the false negative rate of freshness detection by 18–22% compared to stationary storage.

#### 5.6.5. Turkey

For turkey hatching eggs, long-term storage (≥14 days) leads to more pronounced embryonic developmental delays than in chicken eggs, requiring adjusted detection windows. HSI-based fertility detection in turkey eggs achieves 87% accuracy at day 3 of incubation, while VOC analysis has shown potential for early in ovo sexing, with 80% accuracy at day 5 of incubation [42]. Overall, turkey egg detection research remains far less mature than chicken egg research, with very few independent validation studies.

Across all species, models optimized for chicken eggs typically exhibit 20–30% lower accuracy when directly applied to non-chicken species. This highlights the importance of species-specific technology development and the potential value of transfer learning approaches to reduce data collection burdens for understudied species.

### 5.7. Critical Appraisal and Commercialization Failures

From a critical perspective, the in ovo sexing field suffers from three systemic issues that have slowed commercialization. First, there is widespread inconsistency in reported performance: accuracies for the same technology at the same incubation day can vary by 15–25% across studies, driven by differences in genetic strain, sample selection, acquisition protocol, and validation methodology. Many studies train and test models on the same batch of eggs using random split validation, which systematically inflates accuracy estimates compared to cross-batch external validation.

Second, several high-profile commercialization attempts have failed, revealing gaps in real-world viability. One European company developed a Raman spectroscopy-based in ovo sexing system that achieved 95% accuracy in laboratory tests but was abandoned due to prohibitive maintenance costs: the system required daily laser calibration and quarterly optical component replacement, resulting in annual maintenance costs exceeding $500,000 per unit [13]. Another VOC-based detection system showed promising results in controlled laboratory environments but suffered from severe cross-sensitivity to ambient odors in commercial hatcheries, leading to unstable performance and high false positive rates.

Third, embryonic biosafety data remain incomplete and inconsistent across studies. While most research reports no significant effect on hatching rate, some independent studies have found that laser powers above 5 mW can increase embryonic malformation rates by 3–5%, and prolonged light exposure can alter embryonic developmental timing [68]. There is no standardized biosafety testing protocol, making it difficult to compare risks across technologies.

## 6. Algorithmic Challenges and Industrial Implementation

### 6.1. Core Algorithmic and Industrial-Scale Challenges

From a modeling perspective, the primary challenges in hatching egg non-destructive detection are not a lack of algorithms, but rather weak signal strength, high sample heterogeneity, and insufficient independent external validation. SVM and PLS-DA are well-suited for small-to-medium sample spectral modeling, offering the advantages of fewer parameters and relatively interpretable results. RF and D-S fusion approaches are effective for integrating complementary spectral and image information. CNN and deep learning models enable automatic extraction of nonlinear features, but they require significantly larger sample sizes and more rigorous independent testing [24,25,26].

Beyond basic algorithmic performance, three understudied challenges are critical for industrial deployment but rarely addressed in academic research. First, batch effects: systematic signal shifts between eggs from different breeder flocks, age groups, or production seasons can degrade model accuracy by 10–20%, even within the same genetic strain. Most published models are not designed to handle these shifts. Second, model drift: sensor aging, light source degradation, and gradual changes in production conditions cause model performance to decline over time, requiring periodic recalibration that is not accounted for in static laboratory validation. Third, few-shot generalization: commercial hatcheries frequently introduce new genetic strains or change management practices, and models must adapt with minimal new training data—a capability that current detection systems largely lack.

Consequently, the quality of a study cannot be evaluated solely on the basis of “peak accuracy”; it must also be assessed based on sample size, incubation day at detection, training/test set partitioning, genetic strain and eggshell color ranges, and whether hatching rates and chick quality are reported. Models trained and tested on the same batch of eggs often exhibit inflated performance metrics that do not generalize to new production batches or different genetic strains.

### 6.2. Integrated System Architecture for Industrial Deployment

**Figure 4:**
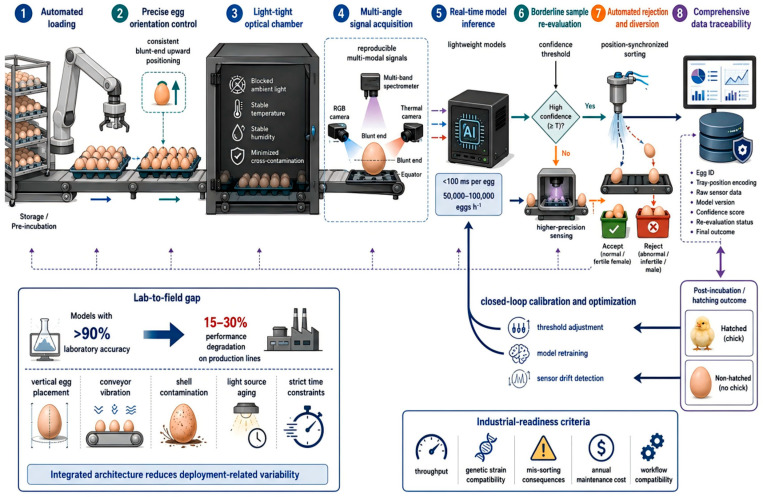
Integrated system architecture for industrial deployment of hatching egg non-destructive detection technologies.

The successful translation of laboratory-validated classification models into commercial hatchery operations relies not on isolated algorithmic performance, but on the seamless integration of sensing hardware, mechanical handling systems, real-time computing infrastructure, and closed-loop data management. Unlike controlled offline experiments that process static, individually positioned samples, industrial hatchery detection operates as a continuous, high-throughput end-to-end workflow where every stage of physical handling directly impacts signal quality and classification reliability. As illustrated in Figure 4, a fully functional hatchery-ready detection system comprises eight interdependent core modules that collectively address the unique constraints of commercial production:

(1) Automated loading module: Receives standard egg trays directly from storage or pre-incubation rooms, ensuring consistent tray alignment and gentle handling.
(2) Precise egg orientation control: Standardizes egg positioning to blunt-end upward orientation, matching commercial incubation practices.
(3) Light-tight optical chamber: Isolates the detection zone from ambient light interference and maintains stable temperature and humidity conditions.
(4) Multi-angle signal acquisition: Integrates synchronized multi-modal sensors at predefined positions to capture consistent, biologically relevant signals.
(5) Real-time model inference: Deploys optimized lightweight models on edge computing devices to process signals within <100 ms per egg.
(6) Borderline sample re-evaluation mechanism: Implements confidence threshold-based decision logic, diverting low-confidence samples for secondary inspection.
(7) Automated rejection and diversion: Activates position-synchronized high-speed pneumatic actuators to divert abnormal, infertile, or male eggs.
(8) Comprehensive data traceability system: Links each egg’s unique identifier to raw sensor data, model version, confidence score, and final hatching outcome.

This integrated architecture directly mitigates the pervasive “lab-to-field gap” in hatching egg detection technologies. As quantified in Figure 4, models that achieve >90% accuracy under controlled laboratory conditions typically experience 15–30% performance degradation when deployed on production lines, primarily due to five unaccounted-for variables: vertical egg placement in commercial trays, conveyor-induced vibration, eggshell surface contamination, gradual light source degradation, and strict per-egg processing time constraints [13]. The standardized handling and controlled measurement environment provided by the eight-module architecture systematically reduces these sources of deployment-related variability.

Crucially, the system incorporates a closed-loop calibration and optimization cycle enabled by the data traceability module. By correlating real-time detection results with post-incubation hatching outcomes, the system can automatically perform dynamic threshold adjustment, periodic model retraining, and early detection of sensor drift. This continuous feedback loop is essential for maintaining stable long-term performance across varying production conditions.

From a cost–benefit perspective, a commercial in ovo sexing system with a capacity of 100,000 eggs per hour typically requires an initial investment of $1.5–2.5 million, with annual maintenance costs of $150,000–300,000. It can save hatcheries $2–4 million annually by reducing incubation energy costs, labor costs for male chick culling, and waste disposal expenses, resulting in a payback period of 1–2 years for large-scale hatcheries [2]. For small-scale hatcheries, however, the high upfront cost remains a significant barrier.

### 6.3. Hierarchical Detection Strategy

A more practical implementation strategy is hierarchical detection. The first tier employs machine vision, thermal imaging, or multi-band Vis-NIR spectroscopy for high-speed primary screening, targeting removal of clearly abnormal, infertile, or low-viability samples. The second tier uses HSI, VOC analysis, OCT, or spectral-image fusion methods for re-evaluation of borderline samples, high-value hatching eggs, or embryos at specific incubation days.

This hierarchical strategy also introduces inherent limitations. First, integrating multiple sensing modalities, mechanical diversion mechanisms, and data synchronization systems raises engineering complexity and failure points. Second, a two-tier system requires additional sensors and sorting actuators, increasing upfront costs by 30–50% compared to single-modality systems. Third, borderline samples require secondary inspection, which can decrease effective processing speed by 10–15% compared to continuous single-pass sorting. Fourth, the economies of scale of hierarchical systems are only realized at processing capacities above 50,000 eggs per hour; for small hatcheries with capacities below 10,000 eggs per hour, the cost–benefit ratio becomes unfavorable.

The industrial suitability of different technical approaches can be evaluated based on four key dimensions: information content, throughput potential, cost, and maintenance complexity, as summarized in Table 3.

### 6.4. Critical Factors for Laboratory-to-Industrial Translation

#### 6.4.1. Detection Timing and Embryonic Biosafety

The timing of detection is the primary determinant of technological value in hatching egg inspection. Pre-incubation freshness screening, if completed prior to loading into incubators, directly reduces ineffective incubation and associated resource waste. Conversely, delaying in ovo sexing to mid-to-late incubation stages diminishes both economic returns and animal welfare benefits, even if classification accuracy improves [49,50]. Therefore, in ovo sexing studies should not only report classification accuracy but also explicitly specify incubation day at detection, per-egg processing time, sample size, eggshell color range, and genetic strain coverage.

For freshness and viability detection, hatching rate, malformation rate, and post-hatch chick quality must be included as critical post-detection validation outcomes. Existing data indicate that most non-contact optical detection procedures do not significantly reduce hatching rate when exposure times are kept below 30 s and laser power is below 5 mW. However, eggshell windowing, prolonged heating, and extended laser exposure have been associated with 3–8% higher embryonic mortality and 2–5% higher malformation rates in some independent studies [68]. All experiments involving such interventions must separately report embryonic safety outcomes.

#### 6.4.2. Signal Interpretability and Cross-Batch Generalizability

The interpretability of detection signals is the key determinant of model cross-batch generalizability. Model predictions are more likely to be reproducible across different production batches and equipment configurations when spectral bands can be explicitly linked to biological processes such as blood absorption, water migration, or changes in yolk or albumen composition, and when machine vision features correspond to vascular development, air cell enlargement, or germinal disk position. In contrast, models that merely incorporate spectral bands, texture features, VOC profiles, or impedance measurements into classifiers without establishing their biological correlation with hatching egg status typically lose stability when applied to different strains, eggshell colors, or storage conditions. Therefore, public sharing of datasets, raw spectra, and images, combined with stratified external validation by incubation day, eggshell color, and genetic strain, provides a more robust measure of method reliability than the peak accuracy achieved in a single experiment [36,52,53].

#### 6.4.3. Stratified Technology and Evidence System

Non-destructive, minimally invasive, and validation technologies must be clearly stratified according to their intended production applications. Molecular and cytological methods provide highly reliable reference standards and help elucidate the biological origins of optical or olfactory signals. However, any technique requiring sampling, eggshell windowing, or disruption of the embryonic environment should not be classified as part of the mainline online non-destructive detection workflow [56,65].

A rational evaluation framework positions each technology within the broader hatchery operational chain: pre-incubation screening prioritizes low cost and high throughput; early viability assessment emphasizes embryonic safety; in ovo sexing prioritizes early detection timing and low mis-sorting cost; re-evaluation focuses on high information content; and mechanistic validation requires reference reliability.

## 7. Conclusions

This review establishes a unifying scientific foundation for non-destructive hatching egg detection: robust, mechanistically interpretable, and industrially integrable signals can be systematically extracted from intra-egg quality variations, embryonic developmental trajectories, and sex-specific metabolic differences. The analysis confirms that hyperspectral imaging, Vis-NIR spectroscopy, thermal imaging, machine vision, VOC analysis, bioimpedance, and OCT all provide valid diagnostic information within well-defined temporal windows, and that multi-modal fusion and advanced machine learning can substantially enhance discrimination of weak early-stage signals.

Critically, the primary bottlenecks to widespread industrial implementation no longer reside in achieving high classification accuracy in isolated laboratory models. Instead, the field’s most intractable challenges lie in simultaneously satisfying commercial hatcheries’ requirements for standardized sample preparation, cross-batch generalizability, rigorous embryonic biosafety assessment, high-throughput online operation, and low-cost equipment maintenance. The integration of pre-incubation screening, in ovo sex identification, and post-hatch outcome feedback into a closed-loop technical pipeline represents a transformative paradigm shift that converts decades of fragmented sensor research into comparable, verifiable, and iteratively improvable industrial equipment solutions.

Moving forward, the field should prioritize two interconnected research imperatives. First, establishment of a comprehensive shared data ecosystem that systematically covers genetic strains, eggshell colors, storage durations, detection days, and corresponding hatching outcomes, to address the current crisis of irreproducible results. Second, development of hierarchical detection architectures that synergize low-cost high-throughput primary screening with high-information-content confirmatory testing, balancing performance, cost, and throughput in a way that no single-modality system can achieve. These advances will guide the field toward a more sustainable and ethical future for global poultry production.

## Figures and Tables

**Table 1:** Summary of technical characteristics, detection windows, and performance metrics of representative pre-incubation freshness detection technologies.

Testing Information	Technical Means	Signal and Modeling Notes
Fertility/infertility	HSI transmittance	Transmitted spectra and germinal-disk signal; LDA/ANN/SVM; accuracy 86.67–92.59%; unincubated eggs
Fertility/hatchability	HSI + relevance vector machine	400–1000 nm spectral-spatial information; recognition rate ~90%; dependent on sample split
Storage freshness	Egg-parameter model	Egg shape, mass and air-cell variation; multivariable regression; controlled conditions
Oxygen respiration	Non-invasive micro-test	Oxygen flux during storage; regression analysis; reported R^2^ ~0.96
Air-cell change	Thermal IR imaging	Thermal contrast from air-cell enlargement; Faster R-CNN; detection mAP up to 99.85%
Vascular viability	Laser speckle contrast imaging	Extra-embryonic blood-flow signal; day 3–5 vascular network observable

**Table 2:** Summary of performance characteristics, detection windows, and validation methodologies of representative embryonic sex detection technologies.

Detection Target	Technical Modality	Representative Performance and Conditions	Detection Window	Main Limitations
Chicken embryo sex	Hyperspectral imaging	Accuracy ~75–82.86% around day 10	Day 10	Weak early signals, significant eggshell color interference
Early chicken embryo sex	Spectroscopy + machine vision fusion	Fusion accuracy 88.00% on day 4; 566 white-shelled eggs	Day 4	Horizontal acquisition does not match production line trays
Chicken embryo sex	Two-wavelength fluorescence spectroscopy	Accuracy ~96% on day 7 of incubation	Day 7	Embryonic effects of optical exposure not fully evaluated
Chicken embryo sex	SPME/GC-MS odor analysis	Distinguishable male and female odor profiles	Day 3–10	Low throughput, pending sensor array validation
Sex verification/control	Molecular methods (PCR, LAMP)	High accuracy, gold standard reference	Day 1–4	Requires sampling, not suitable for online detection

**Table 3:** Multi-dimensional comparison of industrial suitability for major hatching egg non-destructive detection technologies.

Technical Approach	Information Content	Throughput Potential	Cost	Suitable Application Stage
Multi-band Vis-NIR spectroscopy	Medium	High	Low-Medium	Pre-incubation screening, early recheck
Hyperspectral imaging	High	Medium	High	Mechanistic research, high-value batch recheck
RGB/Thermal imaging	Medium	High	Low-Medium	Primary screening of abnormal eggs
VOCs/Gas analysis	Medium	Low-Medium	Medium-High	Metabolic difference exploration
Optical coherence tomography	High	Low	High	Germinal disk localization
Molecular detection	High	Low	Medium	Gold standard validation

## Data Availability

The data presented in this study are available within the article. Further inquiries can be directed to the first author (Qian Yan, yqyq@webmail.hzau.edu.cn).
